# Decreasing Emergency Department Walkout Rate and Boarding Hours by Improving Inpatient Length of Stay

**DOI:** 10.5811/westjem.2017.7.34663

**Published:** 2017-09-18

**Authors:** Andrew W. Artenstein, Niels K. Rathlev, Douglas Neal, Vernette Townsend, Michael Vemula, Sheila Goldlust, Joseph Schmidt, Paul Visintainer, Baystate Patient Progress Initiative Team Members, Michael Albert

**Affiliations:** * University of Massachusetts Medical School-Baystate, Department of Medicine, Division of Infectious Disease, Springfield, Massachusetts; † Baystate Health, Healthcare Quality and Process Improvement, Springfield, Massachusetts; ‡ Baystate Health, Baystate Medical Center, Springfield, Massachusetts; § Baystate Health, Department of Medicine, Springfield, Massachusetts; ¶ University of Massachusetts Medical School-Department of Emergency Medicine, Baystate, Springfield, Massachusetts; || University of Massachusetts Medical School-Baystate, Office of Research, Springfield, Massachusetts

## Abstract

**Introduction:**

Patient progress, the movement of patients through a hospital system from admission to discharge, is a foundational component of operational effectiveness in healthcare institutions. Optimal patient progress is a key to delivering safe, high-quality and high-value clinical care. The Baystate Patient Progress Initiative (BPPI), a cross-disciplinary, multifaceted quality and process improvement project, was launched on March 1, 2014, with the primary goal of optimizing patient progress for adult patients.

**Methods:**

The BPPI was implemented at our system’s tertiary care, academic medical center, a high-volume, high-acuity hospital that serves as a regional referral center for western Massachusetts. The BPPI was structured as a 24-month initiative with an oversight group that ensured collaborative goal alignment and communication of operational teams. It was organized to address critical aspects of a patient’s progress through his hospital stay and to create additional inpatient capacity. The specific goal of the BPPI was to decrease length of stay (LOS) on the inpatient adult Hospital Medicine service by optimizing an interdisciplinary plan of care and promoting earlier departure of discharged patients. Concurrently, we measured the effects on emergency department (ED) boarding hours per patient and walkout rates.

**Results:**

The BPPI engaged over 300 employed clinicians and non-clinicians in the work. We created increased inpatient capacity by implementing daily interdisciplinary bedside rounds to proactively address patient progress; during the 24 months, this resulted in a sustained rate of discharge orders written before noon of more than 50% and a decrease in inpatient LOS of 0.30 days (coefficient: −0.014, 95% CI [−0.023, −0.005] P< 0.005). Despite the increase in ED patient volumes and severity of illness over the same time period, ED boarding hours per patient decreased by approximately 2.1 hours (coefficient: −0.09; 95% CI [−0.15, −0.02] P = 0.007). Concurrently, ED walkout rates decreased by nearly 32% to a monthly mean of 0.4 patients (coefficient: 0.4; 95% CI [−0.7, −0.1] P= 0.01).

**Conclusion:**

The BPPI realized significant gains in patient progress for adult patients by promoting earlier discharges before noon and decreasing overall inpatient LOS. Concurrently, ED boarding hours per patient and walkout rates decreased.

## INTRODUCTION

Healthcare reforms, stimulated by unsustainably escalating costs have led to an accelerating march away from volume-based payment models towards value-based models of payment that incentivize operational efficiencies and patient outcomes.^1^,^2^ High volumes and occupancy rates continue to pose operational challenges for large urban community and teaching hospitals and can negatively impact their ability to deliver high-value care.^3^,^4^ Such operational challenges are generally described in the extant literature under the umbrella terms “patient throughput,” “patient flow,” or “patient progress,” all referring to the movement of patients through a hospital system from admission to discharge.^5^ Fundamentally, patient progress in hospitals is hindered by inpatient and emergency department (ED) capacity and efficiency issues. Much of the existing literature in this arena derives from ED studies and process improvements performed in the focused environment of the operating room;^6^ inpatient studies on patient progress have been performed as well.^7^,^8^

In the ED, bottlenecks along a patient’s path contribute to hindering progress in “input,” i.e., registration and triage; “throughput,” i.e., patient evaluation and management by providers and nursing staff; and “output,” i.e., discharge, transfer, or admission. Some of these barriers lead to “boarding” of inpatients in the ED.^9^ Care of these patients may be delayed, as ED and inpatient teams struggle with the incoming ED volume and the time-sensitive exigencies of patients already occupying inpatient beds. As a result, some patients leave the ED without being seen by a provider (“walkouts”) because of long wait times. Patients who leave against medical advice or who leave before treatment is complete are not included in the “walkouts” category. Each of these outcomes has the potential to adversely affect safety, quality and patient and family experience.^10^

An inverse correlation between patient progress (improving throughput) and patient volumes has been demonstrated in several settings. Patient progress in the ED is significantly impacted by the daily census in the ED and the numbers of ED inpatient admissions.^11^ Investigators have shown an association between ED length of stay (LOS), the number of ED admissions and hospital occupancy rates on inpatient services. Elective admissions for surgical and other procedures may compete directly with ED admissions for a limited number of inpatient beds.^12^ Harrison et al. found that per capita discharge rates, even of patients with longer LOS, were significantly greater during high occupancy periods in the hospital.^13^

Deterioration in patient progress in the ED leads to a higher number of “walkouts,” which is a particularly prevalent challenge in teaching institutions in metropolitan areas.^14^ Several factors have been shown to increase the likelihood of “walkouts”: longer durations of the ED “front-end” process from initial patient presentation to placement in an exam room,^15^,^16^ and ED occupancy (i.e., the number of registered patients divided by the number of licensed ED beds) of greater than 140%.^17^ This is particularly of concern for high-risk patients who occasionally experience adverse outcomes after “walking out” of the ED.^18^ A larger number of these patients re-present to the ED for care within 48 hours as compared with patients who complete evaluation and management during their initial ED presentation.^19^ Institutional revenues may also be impacted negatively.^20^

**Population Health Research Capsule**

What do we already know about this issue?

The efficient movement of patients through their hospitalization (i.e., “patient progress”) from ED admission to hospital discharge contributes significantly to quality and value, thus enhancing population health.

What was the research question?

Would a multi-disciplinary, multifaceted quality and process improvement process—the Baystate Patient Progress Initiative (BPPI)—optimize patient progress by reducing length of stay (LOS) and improving ED walkout rate and boarding hours?

What was the major finding of the study?

The BPPI resulted in a 0.30 day decrease in hospital LOS through multiple tactics. Despite the increase in ED volumes and severity of illness, this effort led to a two-hour reduction in ED boarding hours per patient and a one-third reduction in walkout rates.

How does this improve population health?

The improvements engendered by the BPPI work, based on the extant literature, are likely to improve safety, quality and patient experience—all essential elements of population health. Additionally, the BPPI clearly improved value and accessibility of care.

Boarding of admitted patients in the ED (after the decision to admit has been made), hinders the ability to evaluate, manage, and accept transfer patients in a timely fashion, and thus may lead to ED crowding and ambulance diversion.^21^,^22^ Active bed management using bed management rounds, assigning patients boarding in the ED to inpatient services, and empowering a “bed director” to mobilize additional throughput resources may significantly shorten ED LOS^23^ and may also favorably impact LOS for patients discharged from the ED,^24^,^25^ hospital LOS, ED patient satisfaction scores, and perhaps most importantly, decrease the numbers of patients boarding in the ED.^21^

Several studies have demonstrated an association between ED boarding, hospital LOS and mortality for both hospitalized patients and those directly discharged from the ED.^26^–^29^ The adverse effect on mortality is particularly noteworthy in patients who require critical care. Patients who board for more than six hours in the ED before transfer to the critical care unit have a 4.5% higher inhospital mortality rate than those who board for less than that.^30^ Patient progress also has a significant impact on hospital finances. This may be particularly relevant to large academic and referral hospitals where demand commonly exceeds the supply due to capacity constraints.^31^ The prioritization in bed assignment to elective over ED admissions may delay patient progress by increasing inpatient and ED LOS.^32^ An uneven weekly distribution of elective surgical and procedural admissions may have an adverse effect on functional bed capacity on days with high demand.^33^ “Smoothing” the scheduling of such elective admissions has been demonstrated to have a beneficial effect on patient progress.^6^,^33^

Several groups have reported on initiatives to improve the balance between demand and capacity on inpatient units in acute care hospitals: active “pulling” of admissions from the ED;^34^ multidisciplinary “plan-of-care” daily rounds;^35^,^36^ managing “churn”, i.e., patient movement and bed turnovers across different inpatient units during a single episode of care;^37^ and highly scripted process improvements around the timing and communication process of discharges.^38^,^39^ One other group has reported results from their multidisciplinary, quality improvement initiative around patient “flow.”^8^ The authors addressed process improvements in the ED, inpatient, and support department domains using a physician-led approach with operational support from external consultants. Their outcomes included an improvement in LOS and an increase in the rate of 11 AM discharges.^8^

## METHODS

### Setting

Baystate Medical Center (BMC), a 720-bed and 94 ED-bay, tertiary-care regional, academic medical center serving a population of approximately 850,000 people in western Massachusetts, is the referral center for Baystate Health (BH), a five-hospital, integrated health system serving the region and portions of two neighboring states. By 2012, BMC, the largest and busiest tertiary care referral hospital in the region, was experiencing consistently high ED and inpatient hospital volumes with many operational inefficiencies. Escalating ED walkouts and rising patient LOS on the inpatient units were emblematic of these inefficiencies. During fiscal year (FY) 2013, when the BPPI was initiated, BMC provided care for 109,111 ED visits and 26,335 adult, non-psychiatric and non-obstetric admissions, with a corresponding case-mix index (CMI) of 1.72, which is in the average range for like-size hospitals.

Based on these data and the potential negative impact of these factors, we embarked on a multi-disciplinary, institutional, performance improvement initiative—the Baystate Patient Progress Initiative (BPPI)—with the goal of decreasing ED walkouts and boarding hours, inpatient LOS and increasing the number of patients with written discharge orders before noon.

The chief operating officer/chief physician executive of BH commissioned the BPPI, and senior clinical and administrative leaders from the organization gathered to review hospital performance data and develop a shared vision for systems improvement. This event led directly to the formation of workgroups and selection of group leaders with a planned implementation on March 1, 2014.

The structure of BPPI comprised an executive steering committee that was responsible for the oversight of three discrete operational work teams. Each team was empowered to function autonomously, but the co-leaders were expected to report out metrics at pre-determined intervals. Three operational teams were organized to address various aspects of a patient’s journey and progress through BMC and create long-term capacity to sustain improvements. The scope and activities were focused on the ED and adult Hospital Medicine (inpatient) services. The “ED” and “Right patient, Right bed, Right time” (RRR) teams involved the progression of clinical decision-making and care processes at the most common initial points of patient contact. The “Interdisciplinary Plan-of-Care” (IPOC) team specifically examined the progression of care on the inpatient units.

The initial meeting of each team’s steering group involved a two-day instructional workshop on Lean Sigma methodology, team-building activities, formulation of a problem statement, and delineation of specific activities. Each sub-team then convened to develop individual projects and metrics using a Lean Sigma framework.^40^ The scope of some projects/activities spanned more than one team (e.g. ED and RRR), thus engendering further opportunities for collaborations and spawning the formation of several “hybrid” teams. Large, academic medical centers represent highly complex systems that are often poorly understood, costly, and rife with inefficiencies.^41^ Key systems engineering principles were employed and combined with well-described waste elimination techniques to ensure effectiveness.^41^

### Metrics

The key system-level measures adopted by the executive steering committee for the ED and adult Hospital Medicine (inpatient) services were the following: 1) number of registered ED patients and walkouts per day; 2) number of boarding hours per ED admission; 3) percentage of inpatient discharge orders written before noon; 4) percentage of inpatients on daily IPOC; and 5) inpatient LOS. The measures were calculated and reported as monthly means for the days in each month. The metrics for each team and sub-teams of BPPI are shown in the Table.

### Statistical Analysis

We measured the primary analytic outcomes monthly as either mean daily counts or as percentages over the specified time period. Mean daily counts were computed as the total monthly count divided by the number of days in the month. As these mean daily counts were approximately normally distributed, we analyzed data using parametric testing. Linear trends over time in mean daily volumes or mean daily walkouts were estimated using linear regression. For outcomes measured as percentages, we used generalized linear models, designating the distributional family as binomial and a log link function. Robust standard errors were used in these analyses. All regression slope coefficients are reported with 95% confidence intervals (CI) and p-values. We added trend lines to figures to aid in interpretation. Our investigational review board did not require review of the project since the project was designed for performance improvement.

## RESULTS

### Participants

The BPPI engaged more than 300 direct participants in the work of the teams. Of these participants, 43% represented frontline clinical staff, such as hospital-based physicians, nurses, and patient care technicians. The remainder of the participants was largely divided between other clinical- and non-clinical support staff. Of the total participants, 40% were nurses and 22% were physicians or advanced practice clinicians.

### Volume and Walkouts

**Figure 1:**
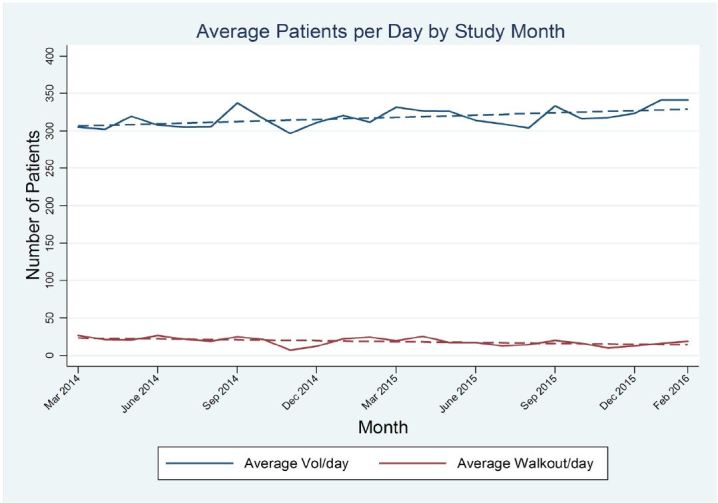
Mean number of registered emergency department patients and walkouts per day. Mean # of Patients /day: coefficient 1.0 (95% CI [0.3,1.7] P < 0.006). Mean Walkouts/day: coefficient −0.4 (95% CI [−0.7, −0.1] P= 0.01.

Over the 24-month study period from March 2014 to February 2016, the mean daily volume increased by about one patient each month over the time period (coefficient: 1.0, 95% CI [0.3, 1.7] P = 0.006) from an estimated 288 patients per day to about 311 per day (Figure 1). These patient volumes make BMC currently the busiest single-site ED in Massachusetts.^42^ Despite progressively rising volumes in the ED, activities of the clinical teams of the BPPI (Table) led to a steady decrease in the monthly mean number of walkouts from approximately 31 patients per day (10.5%) to 21 patients per day (6.7%) over the study period (Figure 1). This resulted in a decrease in the monthly mean number of walkouts by 0.4 registered patients in the ED (coefficient: −0.4; 95% CI [−0.7, −0.1] P= 0.01).

### ED boarding hours per patient

**Figure 2:**
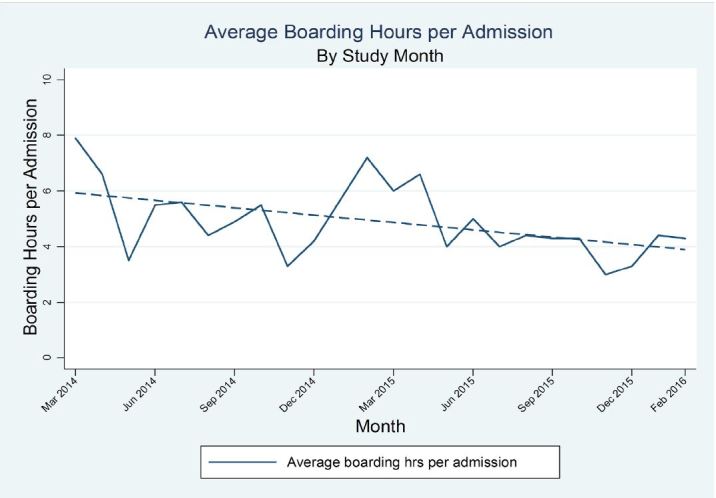
Mean boarding hours per admission: coefficient −0.09 (95% CI [−0.15, −0.02] p=0.007).

ED boarding hours per patient, defined as the duration of time from the decision to admit or assignment to observation while admissions and observation cases are receiving care in the ED, declined through the same 24-month time period from an initial estimate of 7.6 hours to 5.5 hours (coefficient −0.09 hours/month; 95% CI: −0.15, −0.02; p=0.007). This occurred even as admission volumes increased (Figure 2). The literature suggests that boarding hours correlate with wait times and walkout rates.^9^,^16^–^20^ We analyzed incremental changes in the number of boarding hours needed to generate every 1% of left without being seen and found that through the BPPI work we realized progressive, incremental changes from 36 to more than 90 boarding hours needed to generate every 1% of walkouts. These improvements in efficiency in the ED enabled us to reduce walkouts significantly with decreasing boarding hours while experiencing significant increases in ED volumes over time (Figure 1).

### Discharge order entry before noon

**Figure 3:**
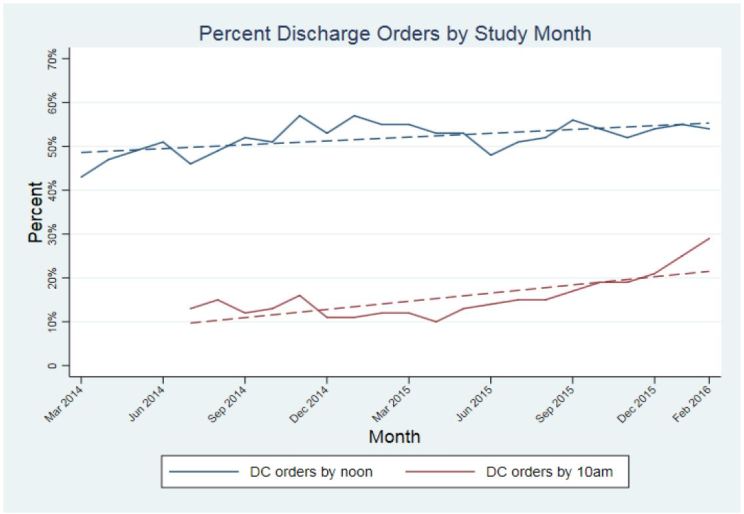
Percent discharge (DC) orders before noon: coefficient 0.5% per month (95% CI [0.3%, 0.8%] p < 0.001).

Improving the timeliness and efficiency of the discharge process was an early focus of the RRR team. Because ED patient arrival patterns at BMC tend to result in peak admission volumes between 1400 hours and 2200 hours, the goal of optimizing discharge order entry by noon, as clinically appropriate, was selected to allow at least two hours for nursing and case management to complete the required documentation and tasks to allow patient egress by early- to mid-afternoon. The rate was approximately 43% at the launch of the initiative. Through the tactics of a focused sub-team of RRR (Table), the rate of discharge order entry before noon progressively rose to 54.1% and was sustained at that level for the duration of BPPI (Figure 3). Discharge orders written before noon increased about 0.5% per month (coefficient 0.5%; 95% CI [0.3%, 0.8%] p < 0.001). The RRR team determined that bed capacity on the inpatient units might be further enhanced by focusing on a subset of patients who could be appropriately discharged earlier in the morning. Even earlier discharge order entry before 10AM was made a priority starting in July 2017. Using similar tactics (Table) to those employed to increase the rate of discharge orders before noon, the team improved the rate of discharge order entry by 10AM by 123% (Figure 3) from 13% to 29%, or an increase of 4.2% per month, (coefficient 4.2%; 95% CI [2.4%, 6.1%] p < 0.001).

### Interdisciplinary plan of care

**Figure 4:**
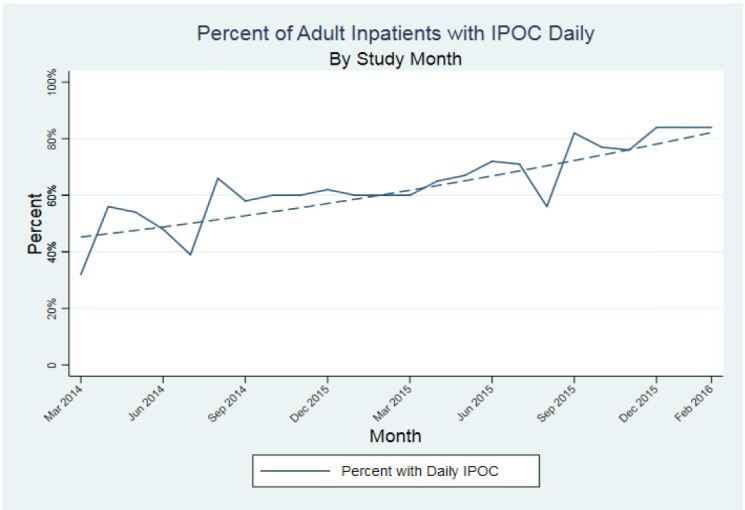
Percent of adult Hospital Medicine inpatients with daily interdisciplinary plan of care (IPOC): coefficient 2.6% (95% CI [2.0%, 3.3%] p <0.001).

The IPOC team was charged with improving performance and patient progress from the time of arrival on the designated inpatient unit through discharge. A primary focus of this team was the development of a process and operational pathway for IPOC bedside rounds.^36^ IPOC rounds were disseminated across all medical and surgical units with the team goal for this activity to involve at least 75% of adult inpatients daily. The percent of adult patients seen on daily IPOC rounds increased significantly by about 2.6% per month (coefficient 2.6%; 95% CI [2.0%, 3.3%]; p < 0.001) from 44% to about 83% overall (Figure 4).

### Inpatient LOS

**Figure 5:**
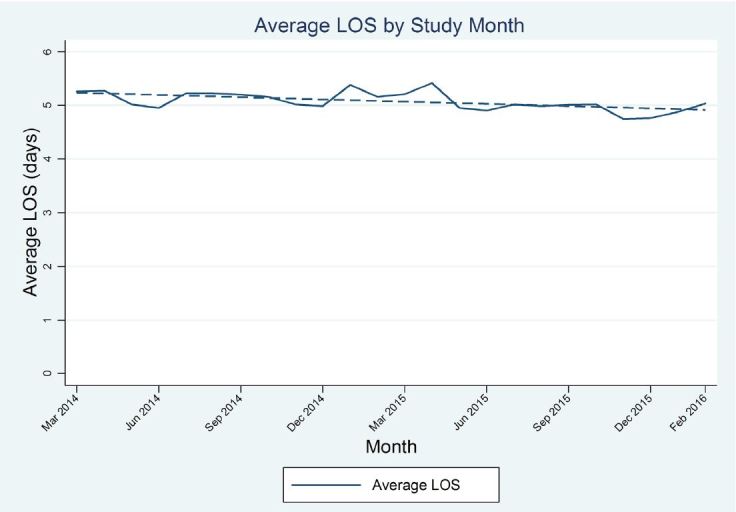
Mean inpatient length of stay (LOS): coefficient: −0.014 (95% CI: −0.023, −0.005; P< 0.005).

An important, overarching, cross-team metric tracked by the BPPI Executive Steering Committee was the diagnosis-related group (DRG)-adjusted, mean LOS for all non-psychiatric, non-obstetric, adult inpatients at BMC. Despite a 9% increase in total annual inpatient volume on the adult Hospital Medicine service over the 24-month time frame after the launch of the initiative, LOS progressively decreased from a baseline of 5.3 days to 5.0 days, representing an absolute improvement of 0.30 days overall (coefficient: −0.014 days /month; 95% CI [−0.023; −0.005]; P< 0.005 [Figure 5]). The mean daily percent of ED patients who were admitted or assigned to observation (27.8%) did not change significantly over this time.

## DISCUSSION

The BPPI, over the two-year charter, achieved several important goals. Through the broad dissemination and adherence to IPOC rounds, which resulted in enhanced communication, coordination, and discharge planning, we progressively and significantly decreased LOS by 0.30 days during the initial phases of BPPI (Figure 5). This created an increase in functional inpatient capacity of 20 open beds per day since we discharged roughly 24,300 patients in the last 12 months of the BPPI [23,000 × 0.3/365 = 20]. In part, this was accomplished by creating inpatient capacity through a focused effort to maximize early discharges as appropriate, yielding a statistically significant, sustained rate of discharge order entry before noon of more than 50% (Figure 3). Additional inpatient bed capacity was created through the coordinated tactics of the IPOC team, targeting daily interdisciplinary bedside rounds to proactively address patient progress milestones during the inpatient component of the hospitalization. Despite progressively accelerating ED (8.3%) and inpatient admission volumes, and a nearly 4% rise in CMI, we concurrently decreased ED boarding hours per admitted patient as well as ED walkout rates by nearly 44% (Figures 1 and 2). While targeted efforts were undertaken and implemented by the ED team to improve ED throughput times, we firmly believe that reducing boarding hours per patient was a very significant contributor to success. Previous literature has notably demonstrated that high ED boarding is a significant contributor to walkouts.^43^

The gains in patient progress achieved through the BPPI become even more meaningful when examined through the prism of other factors that limit bed capacity at our hospital. Because 90% of adult medical beds and 65% of all adult medical/surgical beds are in semi-private rooms, at any given time we are compelled by clinical exigencies to sacrifice capacity by closing beds due to infection control or behavioral issues. Our effective bed utilization (i.e., the number of admitted patients/the number of licensed beds minus closed beds) at these times averaged 103.8%. The literature on bed utilization in acute care hospitals suggests that efficient patient flow is optimal at or below 85%.^44^ Thus, our gains in patient progress occurred despite extraordinary barriers related to constricted inpatient bed capacity. Others have used LOS data from specific patient populations to match bed capacity with demand. Such efforts have resulted in significant reductions in median ED LOS for patients ultimately admitted to adult medicine/surgery units.^45^

A common thread of the extant published literature on patient progress appears to be the segregation of initiatives to specific clinical areas of the hospital such as the ED, operating room, or in some cases the inpatient units. Thus, component parts of the admitted patient’s journey from clinical presentation for care to ultimate disposition and transition out of the hospital may be addressed in various studies by a targeted intervention, but patient progress in the literature is generally not addressed as in this work, as a continuum with multiple “nodes” where quality or process improvement interventions could have an amplified impact. Jweinat et al. reported a collaborative initiative involving several, concurrent process improvement efforts in distinct hospital areas that were physician led but supported by external consultants.^8^ Although the structure and operations of their initiative were distinct from those of the BPPI, several of their outcomes were comparable, thus lending credence to the potential utility of a combination of approaches to these common challenges.

The structure of BPPI is likely to have facilitated the achievement of favorable outcomes. The leaders from each of the five operational teams served on and reported to the BPPI Executive Steering Committee, thus ensuring frequent, direct accountability to each other and allowing information sharing and active contributions across the key teams. The information sharing and communication that occurred in the monthly Executive Steering Committee meetings provided real-time feedback to team leaders and enhanced their ability to adjust tactics with their teams in nimble fashion. Additionally, leaders of the Executive Steering Committee regularly briefed the senior institutional leadership team of BH to ensure that the BPPI continued to be aligned with our enterprise strategic goals and had the resources needed to achieve its goals.

The three clinical operational teams of BPPI (ED, RRR and IPOC) were structurally and functionally organized to follow the patient journey from point of initial clinical contact (the ED for most admissions) to their stay on the inpatient units and subsequent transition from the hospital back to the community. This design was intentional; we believe that it compelled us to address, concurrently and in parallel, many of the variables that affect patient progress in a complex hospital system. Moreover, we believe that such a high level of engagement and participation of clinicians was fundamental to the success of the BPPI.

## LIMITATIONS

Several aspects of our study may limit its generalizability. Because BMC is an academic medical center, we have both “teaching” and “non-teaching” clinical services, each with somewhat distinct operational procedures. Although we addressed both in the BPPI, it is possible that some of our approaches may not apply to non-academic centers. However, because our structure inherently creates additional complexities, we believe our outcomes may potentially underestimate those that could be obtained in a more homogeneous system. Additionally, our institution employs essentially all the inpatient provider staff, including emergency and Hospital Medicine clinicians. This facilitates goal alignment of individuals and teams with those of the system, thus enabling the execution of such an enterprise-wide project as the BPPI. Due to the design as a two-year performance improvement project, we cannot claim to have demonstrated a “cause-and effect” relationship between inpatient LOS, ED boarding and ED walkouts although common sense and logic would argue that an association exists. Certainly, previous literature has demonstrated that ED boarding is a significant driver of walkouts.^43^ Moreover, it is possible that the Hawthorne effect contributed to the beneficial outcomes. As a systemwide initiative, it was not possible to “blind” providers, nurses and other staff with direct patient-care responsibilities to its purpose. The BPPI consumed significant institutional resources, mainly in the form of participant time; however, we did not attempt to estimate the costs, and therefore cannot address the relative cost effectiveness of this initiative.

## CONCLUSION

Through the implementation of a broad, cross-disciplinary, multifaceted, system improvement initiative, we successfully effected significant improvements in patient progress at our institution. These improvements are evidenced by clinically and statistically significant declines in inpatient LOS related to early hospital discharge order entry and multidisciplinary discharge planning. Concomitantly, the ED walkout rate decreased significantly and ED boarding hours remained stable per patient in the face of progressively rising volumes. The BPPI approach may be useful to inform others in healthcare struggling with similar patient progress challenges.

## Figures and Tables

**Table:** Baystate Patient Progress Initiative operational team projects/activities, tactics, and metrics.

Team	Projects/activities	Tactics	Metrics
ED	Staffing to demand Discharge – green light to leave ED Transportation – request to leave ED Triage – entry to assessment	Analyze historical arrival patterns. Set productivity benchmarks. Change schedules Develop discharge standard work Develop discharge standard work. Align staffing to demand Develop Triage standard work. Re-align RN role combined with clerk	Each shift is staffed to expected historical demand Reduce time from ready to leave to discharge Reduce time from bed assign to leave ED Reduce wait time and time to full assessment
RRR	Discharge efficiency Gray Zone Alternate sites of care Early initiation of plan-of-care Geographic rounding Geographic admitting	Highlight discharge orders at hospitalist huddles Assign 2 senior clinicians to ED for 1 week each. Collaborate with post-acute teams on building care models Hospitalist Medicine collaborative team to create capacity to see patients in ED Create schedules to align Hospitalists with nursing units	Define the care team. Set time to round as a team. Build script and run in a simulated environment Calculate expected discharges based on historical data. IPOC team to identify expected discharges for tomorrow. Map out flow of discharge process. Set discharge windows. Develop white boards collaboratively with patients and ancillary staff Collaborative work with IT/Informatics to build IPOC in EMR. Develop My-Plan that is presented daily to patients Move beyond pilot units
IPOC	Collaborative rounding Discharge prediction Day of discharge Patient information boards IPOC components in EMR My-plan for patients Medicine spread H&V spread Surgical spread	Define the care team. Set time to round as a team. Build script and run in a simulated environment Calculate expected discharges based on historical data. IPOC team to identify expected discharges for tomorrow. Map out flow of discharge process. Set discharge windows. Develop white boards collaboratively with patients and ancillary staff Collaborative work with IT/Informatics to build IPOC in EMR. Develop My-Plan that is presented daily to patients Move beyond pilot units	% Pts with IPOC every day % discharge accuracy % Pts discharged within 2 hours of order % Pts with boards completed daily % Pt with My-Plan daily # of units following standard work

*ED,* emergency department; *RRR*, Right patient, Right bed, Right time; *IPOC*, interdisciplinary plan of care; *H&V*, heart & vascular; *EMR*, electronic medical record; *IT*, information technology.
